# Taking action to advance the study of race and ethnicity: the Women’s Health Initiative (WHI)

**DOI:** 10.1186/s40695-021-00071-6

**Published:** 2022-01-04

**Authors:** Lorena Garcia, Shawna Follis, Cynthia A. Thomson, Khadijah Breathett, Crystal Wiley Cené, Monik Jimenez, Charles Kooperberg, Kamal Masaki, Electra D. Paskett, Mary Pettinger, Aaron Aragaki, Peggye Dilworth-Anderson, Marcia L. Stefanick

**Affiliations:** 1 UC Davis School of Medicine, Department of Public Health Sciences, Davis, CA USA; 2 Stanford Prevention Research Center, Department of Medicine, Stanford University, Stanford, CA USA; 3 Department of Nutritional Sciences, University of Arizona, Tucson, AZ USA; 4 Division of Cardiology, College of Medicine, University of Arizona, Tucson, AZ USA; 5 UNC School of Medicine, Department of Medicine, Chapel Hill, NC USA; 6 Division of Women’s Health and Division of Preventive Medicine, Brigham and Women’s Hospital, Harvard Medical School, Boston, MA USA; 7 Public Health Sciences Division, Fred Hutchinson Cancer Research Center, Seattle, WA USA; 8 Department of Geriatric Medicine, John A. Burns School of Medicine, University of Hawaii, Honolulu, HI USA; 9 College of Public Health, The Ohio State University, Columbus, OH USA; 10 Department of Health Policy and Management, Gillings School of Global Public Health at UNC, Chapel Hill, NC USA; 11 Department of Obstetrics & Gynecology, Stanford University, Stanford, CA USA

**Keywords:** Structural racism, Women’s health, Social determinants of health

## Abstract

“Race” and “ethnicity” are socially constructed terms, not based on biology - in contrast to biologic ancestry and genetic admixture - and are flexible, contested, and unstable concepts, often driven by power. Although individuals may self-identify with a given race and ethnic group, as multidimensional beings exposed to differential life influencing factors that contribute to disease risk, additional social determinants of health (SDOH) should be explored to understand the relationship of race or ethnicity to health. Potential health effects of structural racism, defined as “the structures, policies, practices, and norms resulting in differential access to goods, services, and opportunities of society by “race,” have been largely ignored in medical research. The Women’s Health Initiative (WHI) was expected to enroll a racially and ethnically diverse cohort of older women at 40 U.S. clinical centers between 1993 and 1998; yet, key information on the racial and ethnic make-up of the WHI cohort of 161,808 women was limited until a 2020–2021 Task Force was charged by the WHI Steering Committee to better characterize the WHI cohort and develop recommendations for WHI investigators who want to include “race” and/or “ethnicity” in papers and presentations. As the lessons learned are of relevance to most cohorts, the essence of the WHI Race and Ethnicity Language and Data Interpretation Guide is presented in this paper. Recommendations from the WHI Race and Ethnicity Language and Data Interpretation Guide include: Studies should be designed to include all populations and researchers should actively, purposefully and with cultural-relevance, commit to recruiting a diverse sample; Researchers should collect robust data on race, ethnicity and SDOH variables that may intersect with participant identities, such as immigration status, country of origin, acculturation, current residence and neighborhood, religion; Authors should use appropriate terminology, based on a participant’s self-identified “race” and “ethnicity”, and provide clear rationale, including a conceptual framework, for including race and ethnicity in the analytic plan; Researchers should employ appropriate analytical methods, including mixed-methods, to study the relationship of these sociocultural variables to health; Authors should address how representative study participants are of the population to which results might apply, such as by age, race and ethnicity.

**Supplementary Information:**

The online version contains supplementary material available at 10.1186/s40695-021-00071-6.

## Introduction

In the summer of 2020, the Women’s Health Initiative (WHI) Steering Committee assembled a Race and Ethnicity Task Force to evaluate the strengths and limitations of the WHI race and ethnicity data and to provide guidance on language and data interpretation of WHI analyses and manuscripts. We present the WHI Race and Ethnicity Language and Data Interpretation Guide in this paper, as a means to support advancements in the study of race and ethnicity in public health research.

It should be well recognized that “race” and “ethnicity” are each socially constructed terms that are not rooted in biology [1–3]; in fact, a biological basis for race has been definitively debunked in the scientific literature [ 4–14]. In contrast to, but not totally independent of, biologic ancestry and genetic admixture, “race” and “ethnicity” are flexible, unstable and contested concepts, often driven by power (political, financial, etc.) [4–14]. Ethnicity, the state of belonging to a social group that has a common national or cultural tradition [15], can include people of all races. Neither term was developed to inform health or biologic research; however, structural racism patterns differential access to social determinants of health (SDOH) for racial and ethnic groups, which leads to health disparities [16]. In fact, the historical and social contexts of race and ethnicity, described as structural racism, are well documented [4, 5, 16]. Structural Racism is apparent in U.S. economic and social policies that influence the lived experiences of persons of different racial and ethnic groups [17, 18], which in turn, impact health.

Unfortunately, the effects of structural racism, defined as “the structures, policies, practices, and norms resulting in differential access to the goods, services, and opportunities of society by ‘race’” [19], have been largely ignored in medical research. Calls for action to address structural racism and related social determinants of health as fundamental drivers of health disparities [20] require a reconfiguration of conceptual frameworks and a revision of how scientific journals report racial and ethnic disparities [21].

The AMA Manual of Style committee has revised the entire subsection on race and ethnicity reporting [22]. In addition, this committee states the following “inclusive language supports diversity and conveys respect”, whereas, “language that imparts bias toward or against persons or groups on characteristics or demographics” perpetuates misinformation and must be avoided [23]. Terms that might have been considered “standard” in the past but are regarded as unacceptable by a large proportion of the public today, such as “negro” (*which was dropped from the 2020 Census)*, “colored”, “oriental”, “Asiatic”, and “Caucasian”, among others, should be avoided. In fact, Flanagin et al state that the general term “minorities” should also be avoided when describing groups or populations, and although they recommend that one specify “racial or ethnic minority groups”, and state that other terms such as “underserved groups or underrepresented populations” may be used, provided the categories of individuals included are defined, and that “marginalized groups” can be suitable in certain contexts if rationale is provided [22, 23], these terms may not be acceptable to a large segment of the population. Referring to any race or ethnicity as “non-White” is clearly inappropriate, as is the nonspecific group label “other”, unless it was a prespecified formal category in a database or research instrument, in which case, categories included in “other” groups should be defined and reported. Furthermore, combining specified groups as “other”, for the purpose of increasing statistical power to make a comparison with a larger specified group, requires clear scientific rationale and justification or should not be done.

The term “women” was used in the WHI to designate individuals who were assigned female at birth and identified as a woman at the time of the study. While the authors acknowledge gender as a social construct and the exclusion of transgender women and gender non-conforming people within WHI, the term “women” will be used throughout this article consistent with the original use of the term in WHI.

## The Women’s health initiative (WHI)

During the 1980s, it became increasingly apparent that health research had disproportionately focused on males, and White people, leading to widespread exclusion of women from clinical trials, as well as top biomedical research ranks, so that key questions regarding preventive measures concerning women’s health were generally unanswered [24]. The Office of Research on Women’s Health (ORWH) was established in 1990 to address the health inequities faced by women [25] and set the stage for the creation and evolution of the Women’s Health Initiative (WHI) [26], which was launched in 1991 in response to NIH policy (made federal law in 1993) for equitable inclusion and retention of women, race, and ethnicity groups [27].

**Fig. 1:**
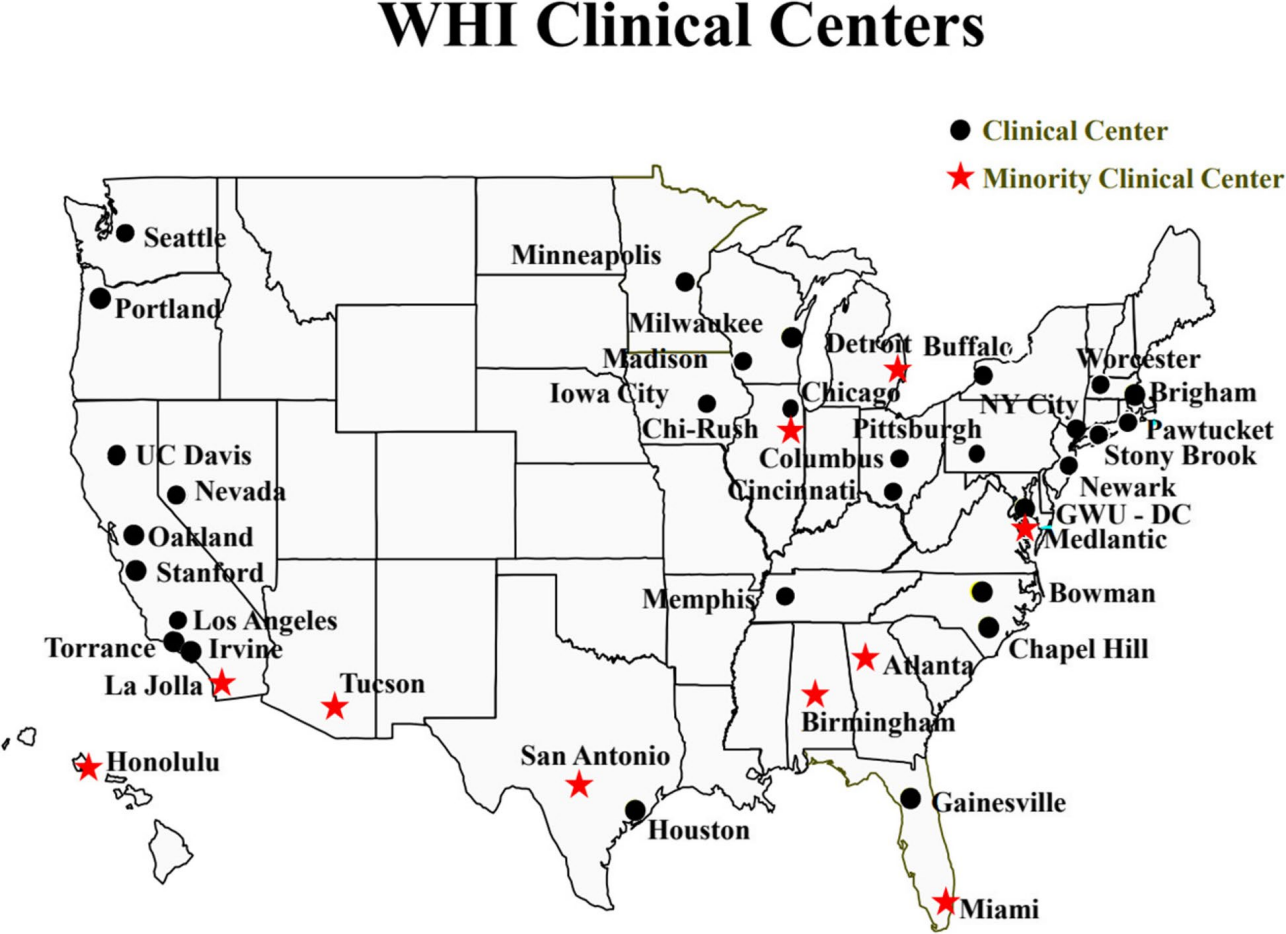
WHI Clinical Centers

WHI set out to become a landmark study of key health issues affecting mid-life to older women (ages 50–79), with a strong commitment for equitable inclusion and retention of race and ethnicity groups historically underrepresented in research, by enrolling at least 20% of the cohort from the following specified racial and ethnic groups: “Native American” (e.g. American Indian/Alaska Native), Asian-American/Pacific Islander *(originally announced as a combined category),* African-American, and Hispanic [26, 28]. To attain the goal of having at least 20% of the WHI participants identify as one of the four specified racial or ethnic groups, 10 of a total of 40 U.S. WHI clinical centers (CC) were designated as “minority recruitment centers “on the basis of their history of interaction with and access to large numbers of women in at least one of the four targeted groups. Each of these 10 centers had the goal of enrolling at least 60% of their participants from these groups (see Fig. 1, U.S. map with the location of WHI clinical centers), while the other 30 WHI CCs were expected to recruit as many women from these historically underrepresented race and ethnicity groups as they could.

Postmenopausal women aged 50–79 were recruited between 1993 and 1998 by the 40 WHI CCs to participate in at least one of two randomized, controlled clinical trials (RCT) of menopausal hormone therapy or a low-fat dietary pattern, with the opportunity to join a third RCT of calcium/vitamin D supplementation a year after enrollment, or the WHI observational study (OS), with all trials and the OS designed to end in 2005 [29]. A total of 161,808 women enrolled in either the Clinical Trial (CT; *N* = 68,132) or OS (*N* = 93,676) WHI components. All WHI participants who were still active in 2005 were invited to reconsent to continued CT or OS follow-up by their respective WHI clinical centers through 2010, at which time all participants were invited to consent to ongoing follow-up in the WHI Extension Study (WHI-ES) through four designated WHI Regional Centers (Northeast, South, Midwest, West) and/or the WHI Clinical Coordinating Center at the Fred Hutchinson Cancer Research Center, Seattle, Washington.

Knowledge contributions from the WHI in relation to the prevention of cardiometabolic diseases, breast, colon and other cancers, fractures, cognitive function and a broad range of other health issues among postmenopausal and older women are well substantiated [29–37]. Yet, efforts to address persistent health disparities along the intersection of race, ethnicity, and age in women’s health have yet to be adequately achieved.

## Race and ethnicity methods in the Women’s health initiative (WHI)

The baseline WHI form asked participants to “describe your race or ethnic group” and “if of mixed blood, which group do you identify with most?” Six categories were offered: (1) American Indian or Alaska Native; (2) Asian or Pacific Islander *(ancestry is Chinese, Indo- Chinese, Korean, Japanese, Pacific Islander, Vietnamese)*; (3) Black or African-American *(not of Hispanic origin);* (4) Hispanic/Latino *(ancestry is Mexican, Cuban, Puerto Rican, Central American, or South American);* (5) White *(not of Hispanic origin);* and, (“8”) Other *(Specify).* As WHI recruitment was nearly complete in 1997, no changes were made to baseline forms when the NIH made two modifications to the collection of race and ethnicity data to (1) separate “Asian” from “Native Hawaiian or Other Pacific Islander”, and (2) change the term “Hispanic” to “Hispanic or Latino”, thereby clearly distinguishing five race categories and two ethnic categories, “Hispanic or Latino” or “Not Hispanic or Latino” [38].

The 10 “minority recruitment sites” sites averaged 43% enrollment of women in the targeted ethnic and racial groups, with only one site (Honolulu, Hawaii) achieving the ≥60% enrollment goal [38]; however, considerable efforts to achieve the overall 20% study goal were put forth by the other 30 sites, which averaged 7.5% racial and ethnic target enrollments. Thus, 18.5% of the women who enrolled in the WHI clinical trials and 16.7% of the women who joined the OS identified as one of the four targeted race or ethnic groups. [“Other” was checked by 1849 participants and 413 participants left the question blank.]

When recruitment strategies were evaluated [28], the use of population-appropriate recruitment materials and strategies was cited as a key driver of diversity in enrollment, including culturally-relevant approaches, such as the *Embajadoras*-led program [39]. Accessibility to a dense target population was also important. For example, the WHI CC in New York City outperformed half of the “minority recruitment sites,” with 37.7% enrollments from targeted race and ethnicity populations.

In 2003, a WHI Special Populations Advisory Committee led an effort to collect new self-identified race and ethnicity data from active participants, using U.S. 2000 Census categories [28]. Participants were asked to identify both their ethnicity and race in two separate questions.

First, Ethnicity: Are you “Spanish/Hispanic/Latino”? Mark (0) “No” box if not Spanish/Hispanic/Latino; (1) Yes, Mexican, Mexican American, or Chicano; (2) Yes, Puerto Rican; (3) Yes, Cuban; (4) Yes, Other Spanish/Hispanic/Latina.

Then, Race: “What is your race? Mark one or more races to indicate what you consider yourself to be: (1) White; (2) Black, African American, Negro; (3) American Indian or Alaska Native; (4) Asian Indian; (5) Chinese; (6) Filipino; (7) Japanese; (8) Korean; (9) Vietnamese; (10) Other Asian; (11) Native Hawaiian; (12) Guamanian or Chamorro; (13) Samoan; (14) Other Pacific Islander; (15) Some other race. *[Note that Asian and Pacific Islander subgroups were presented in the 2000 Census as separate race categories, rather than combining subgroups presented in #4-#10 as “Asian” and in #11-#14 as “Pacific Islander”.]*

**Fig. 2:**
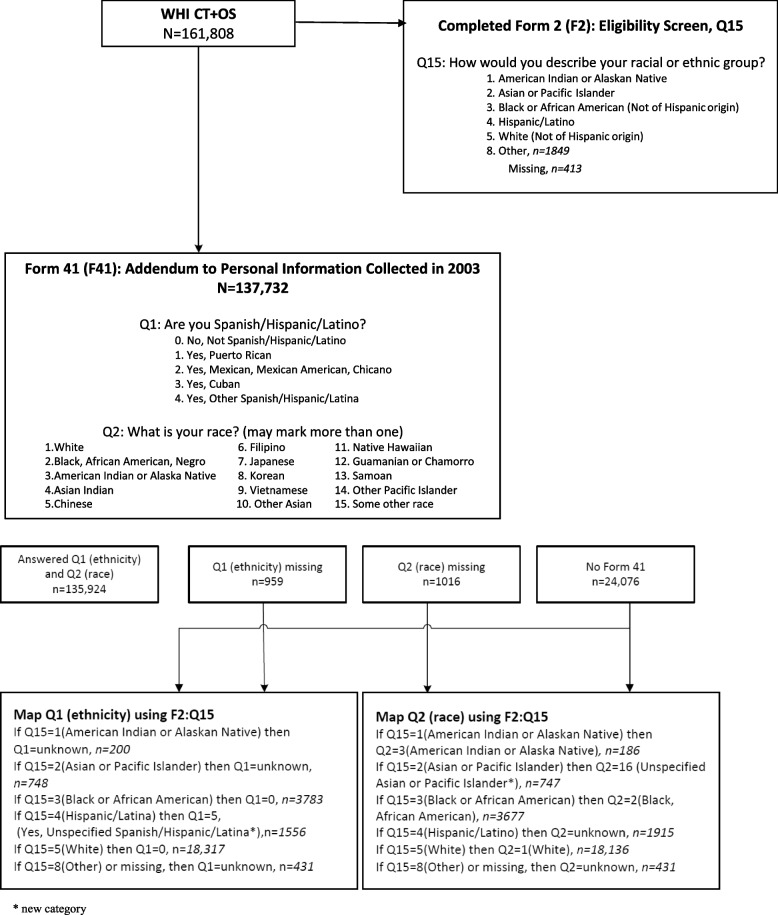
Diagram of Mapping Algorithm

The WHI Race and Ethnicity Task Force (WHI R&E TF) recommended that WHI apply the 2003 (self-identified) categories to the baseline (1993–1998) categories, using a mapping algorithm which is presented as a diagram in Fig. 2. WHI investigators have been instructed to apply these revised race and ethnicity data in future analyses, unless papers are focusing on genetic ancestry or admixture, or if authors have good scientific justification. This has enabled WHI to generate a WHI Cohort Ethnic and Racial Distribution table that conforms to current NIH requirements which was not previously available (Table 1). This activity also enabled WHI investigators to get more detailed information on Hispanic/Latina ethnic subgroups, as well as Asian and Pacific Islander subgroups and multi-racial identities of WHI participants (Table 2).

**Table 1:** NIH Enrollment Table based on WHI mapped Form 41 data

Racial Categories	Ethnic Categories									Total
	Not Hispanic or Latino			Hispanic or Latino			Unknown/Not Reported Ethnicity			
	Female	Male	Unknown/ Not Reported	Female	Male	Unknown/ Not Reported	Female	Male	Unknown/ Not Reported	
American Indian/ Alaskan Native	292	0	0	53	0	0	195	0	0	540
Asian	3216	0	0	60	0	0	749	0	0	4025
Native Hawaiian or Other Pacific Islander	119	0	0	18	0	0	0	0	0	137
Black or African American	14,166	0	0	160	0	0	1	0	0	14,327
White	133,321	0	0	4300	0	0	7	0	0	137,628
More than one Race	1662	0	0	211	0	0	7	0	0	1880
Unknown or Not reported	341	0	0	2510	0	0	420	0	0	3271
**Total**	153,117	0	0	7312	0	0	1379	0	0	161,808

Coding instructions

1). Column categories: use Form 41 imputed Question 1 (Ethnicity); combine ‘Yes, Puerto Rican’, ‘Yes, Mexican, Mexican American, or Chicano’, ‘Yes, Cuban’ and

‘Yes, other Spanish/Hispanic/Latino’ into ‘Hispanic/Latino’.

Row categories: count the number of race categories marked in Question 2 (Race); If number of race categories is greater than one, category = ‘More than one race’; else if number of race categories equals one, use categories for American Indian/Alaskan Native, White, Black or African American as is, and create aggregated categories for Asian = Asian Indian or Chinese or Filipino or Japanese or Korean or Vietnamese or Other Asian, and Native Hawaiian or Pacific Islander = Native Hawaiian or Guamanian/Chamorro or Samoan or Other Pacific Islander.

**Table 2:** Frequency of race and ethnicity categories before and after application of mapping algorithm

*N* = 161,808	As collected on Form 41 or Form 2 N	Mapped value after algorithm application N
**Ethnicity: Spanish/Hispanic/Latino**		
No, Not Spanish/Hispanic/Latino	131,017	153,034
Did not complete 2003 WHI Form/White or Black on baseline WHI Form	22,017	
Yes, Puerto Rican	779	779
Yes, Mexican, Mexican American, Chicano	2693	2693
Yes, Cuban	396	396
Yes, Other Spanish/Hispanic/Latina	1888	1888
Yes, Unspecified Spanish/Hispanic/Latina (Did not complete 2003 WHI Form/Hispanic on baseline WHI Form)	1556	1556
Unknown (Did not complete 2003 WHI Form/Not White/Black/Hispanic on baseline Form)	1379	1379
Unknown (Did not complete2003 or baseline Forms)	83	83
**Total**	**161,808**	**161,808**
**Race:**		
**One reported race**	134,836	157,582
White	119,492	137,628
Black, African American, or Negro	10,650	14,327
American Indian or Alaska Native	354	540
Asian (combining #4–10 from 2003 Form)	3278	4025
Asian Indian	*83*	*83*
Chinese	*747*	*747*
Filipino	*321*	*321*
Japanese	*1962*	*1962*
Korean	*91*	*91*
Vietnamese	*10*	*10*
Other Asian	*64*	*64*
Unspecified Asian (Did not complete 2003 Form/Asian or Pacific Islander, baseline form)	*747*	*747*
Pacific Islander (#11–15 from 2003 Form)	137	137
Native Hawaiian	*97*	*97*
Guamanian or Chamorro	*10*	*10*
Samoan	*2*	*2*
Other Pacific Islander	*28*	*28*
Some other race	925	925
**More than one race**	1880	1880
Unknown (Did not complete 2003 /White, Black, Asian/ PI, American Indian/Alaskan Native on baseline form)	24,661	In one of above categories
Unknown (Did not complete 2003 Form/ Hispanic or Other on baseline form)	2264	2346
Unknown (Did not complete2003 or baseline Forms)	82	
**Total**	**161,808**	**161.808**

One of the driving forces for this effort was the desire to determine how representative of the U.S. population of women, aged 50–79 the WHI was, with respect to race and ethnicity, at baseline. The R & E TF laid out Ethnicity and Race by 5 year age groups for these new categories, in juxtaposition with the 1995 US Census, the latter of which combined Asian and Pacific Islander women (Table 3). When considering total U.S. data for this 30-year age range, the proportion of White WHI participants (86.0%) was slightly lower than the proportion of White U.S. women in 1995 (86.8%), whereas the proportion of Black/African American WHI participants was lower (8.9%) than the U.S. population (9.9%) The age distributions also differed between these groups, with a much higher percent of younger (midlife-aged, i.e. aged 50–64 years) Black women and a lower percent of older (65–79 years) Black women than the U.S. population (Table 3). This was likely a consequence of the WHI decision to stop enrolling White women aged below 55 years in 1996 and below age 60 in 1997, when the prespecified proportion of women by age group, i.e. 10% for ages 50–54 years and 20% for ages 55–59, had been achieved, whereas recruitment of Hispanic women and women of the targeted racial groups continued to the end of the recruitment period in 1998. As seen in Table 3, WHI enrolled a lower proportion of American Indian/Alaska Native women aged 50–79 than resided in the U.S. in 1995, and a higher proportion of older Asian/Pacific Islanders; whereas, with the exception of women ages 50–54 years, the proportion of WHI participants who identified as Hispanic/Latina was substantially lower than the 1995 Census reported for women aged 50–79 years.

**Table 3:** Race and ethnicity (Form 41 imputed) by age groups of WHI Participants at Baseline (1993–1998) compared with the US Census 1995 population estimates for women

US 1995^**4**^	***Ethnicity***	***Race***							
	*Spanish/ Hispanic/* *Latino*	*Black/African American*	*American Indian/Alaska* *Native*	*Asian/ Pacific Islander*		*White*			
Total, %	5.9%	9.9%	0.6%	2.7%		86.8%			
Age, %									
*50 to 54 years*	7.1%	10.9%	0.7%	3.3%		85.2%			
*55 to 59 year*	7.0%	11.1%	0.7%	3.1%		85.2%			
*60 to 64 years*	6.4%	10.6%	0.6%	2.9%		85.9%			
*65 to 69 years*	5.6%	9.7%	0.5%	2.6%		87.3%			
*70 to 74 years*	4.6%	8.3%	0.4%	2.1%		89.2%			
*75 to 79 year*	3.9%	8.0%	0.4%	1.6%		90.0%			
**WHI Baseline**	***Ethnicity***^***1***^	***Race***							
**N = 161,808**	*Spanish/ Hispanic/ Latino*	*Black/African American*	*American Indian/Alaska Native*	*Asian*^*2*^	*Pacific Islander*^*3*^	*White*	*Unknown*	*Some Other Race*	*Two or more races*
Total, N (%)	7312 (4.5%)	14,327 (8.9%)	540 (0.3%)	4025 (2.5%)	137 (0.1%)	137,628 (85.1%)	2346 (1.4%)	925 (0.6%)	1880 (1.2%)
Age, %									
*50 to 54 years*	7.8%	12.4%	0.6%	2.9%	0.2%	78.9%	2.4%	1.1%	1.6%
*55 to 59 year*	5.9%	10.3%	0.4%	2.4%	0.1%	83.1%	1.7%	0.8%	1.3%
*60 to 64 years*	4.5%	9.7%	0.3%	2.3%	0.1%	84.4%	1.5%	0.5%	1.2%
*65 to 69 years*	3.4%	7.1%	0.3%	2.4%	0.1%	87.7%	1.0%	0.4%	1.0%
*70 to 74 years*	2.6%	6.2%	0.3%	2.6%	0.0%	88.6%	1.1%	0.3%	0.9%
*75 to 79 year*	2.2%	6.4%	0.2%	2.8%	0.0%	88.3%	1.1%	0.3%	0.9%

1. Includes Puerto Rican, Mexican, Mexican American, or Chicano, Cuban and other Spanish/Hispanic/Latino

2. Includes Asian Indian or Chinese or Filipino or Japanese or Korean or Vietnamese or Other Asian

3. Includes Native Hawaiian or Guamanian/Chamorro or Samoan or Other Pacific Islander

4. Source: Day, Jennifer Cheeseman, Population Projections of the United States by Age, Sex, Race, and Hispanic Origin: 1995 to 2050, U.S. Bureau of the Census, Current Population Reports, P25–1130, U.S. Government Printing Office, Washington, DC, 1996

Retention of WHI participants has differed by race and ethnicity, such that as of September 2019, with re-consenting required at two time points (2005 and 2010), 89.1% of participants in the current WHI Extension Study cohort, now aged 70 years and older, identified as White at baseline, while the percent of Black/African American had dropped (from 8.9%) to 6.3%, despite their younger baseline age, and the proportion of Hispanic/Latina participants dropped (from 4.5%) to 3.4% (Table 4). Yet, according to 2019 Census estimates for women ages 70 and over, 9.7% of the U.S. population identified as Black/African American and 8.4% identified as Hispanic/Latina. The current WHI analysis of factors related to this lower retention of Hispanic/Latina, Black, Asian, and Native American/Alaska Native women over the nearly 25 years of follow-up is focusing on social determinants of health and structural racism and bias in the context of long-term participation in the study.

**Table 4:** Race and ethnicity (Form 41 imputed) of WHI Extension Study Participants in 2019 compared with the US Census 2019 population estimates for women

US 2019^**4**^	***Ethnicity***	***Race***							
	*Spanish/ Hispanic/ Latino*	*Black/African American*	*American Indian/Alaska Native*	*Asian*	*Pacific Islander*	*White*	*Two or more races*		
Total, %	8.4%	9.7%	0.7%	4.6%	0.1%	84.0%	0.9%		
Age, %									
*70 to 74 years*	8.6%	10.2%	0.8%	4.9%	0.1%	83.0%	1.0%		
*75 to 79 year*	8.4%	9.7%	0.7%	4.5%	0.1%	84.1%	0.9%		
*80 to 84 years*	8.6%	9.6%	0.6%	4.5%	0.1%	84.3%	0.8%		
*85 and over*	7.7%	8.7%	0.5%	4.4%	0.1%	85.6%	0.7%		
**WHI 2019**	***Ethnicity***^***1***^	***Race***							
***N*** **= 67,140**	*Spanish/ Hispanic/ Latino*	*Black/African American*	*American Indian/Alaska Native*	*Asian*^*2*^	*Pacific Islander*^*3*^	*White*	*Unknown*	*Some Other Race*	*Two or more races*
Total, N (%)	2302 (3.4%)	4247 (6.3%)	150 (0.2%)	1394 (2.1%)	49 (0.1%)	59,819 (89.1%)	284 (0.4%)	362 (0.5%)	835 (1.2%)
*Age, %*									
*70 to 74 years*	244 (6.6%)	396 (10.6%)	18 (0.5%)	130 (3.5%)	9 (0.2%)	3028 (81.3%)	31 (0.8%)	50 (1.3%)	64 (1.7%)
*75 to 79 year*	733 (4.3%)	1278 (7.5%)	55 (0.3%)	376 (2.2%)	15 (0.1%)	14,929 (87.2%)	97 (0.6%)	115 (0.7%)	253 (1.5%)
*80 to 84 years*	660 (3.3%)	1303 (6.5%)	42 (0.2%)	375 (1.9%)	14 (0.1%)	17,914 (89.2%)	82 (0.4%)	99 (0.5%)	259 (1.3%)
*85 to 89 years*	426 (2.8%)	808 (5.3%)	21 (0.1%)	310 (2.0%)	8 (0.1%)	13,852 (90.6%)	55 (0.4%)	60 (0.4%)	168 (1.1%)
*90 to 94 years*	198 (2.3%)	375 (4.4%)	10 (0.1%)	158 (1.8%)	3 (0.0%)	7936 (92.2%)	16 (0.2%)	39 (0.3%)	77 (0.9%)
*Over 95 years*	41 (1.8%)	87 (3.8%)	4 (0.2%)	45 (1.9%)	0	2160 (93.0%)	3 (0.1%)	9 (0.4%)	14 (0.6%)

1. Includes Puerto Rican, Mexican, Mexican American, or Chicano, Cuban and other Spanish/Hispanic/Latino

2. Includes Asian Indian or Chinese or Filipino or Japanese or Korean or Vietnamese or Other Asian

3. Includes Native Hawaiian or Guamanian/Chamorro or Samoan or Other Pacific Islander

4. Source: US Census Bureau, Population Division. Annual Estimates of the Resident Population by Sex, Age, Race, and Hispanic Origin for the United States: April 1, 2010 to July 1, 2019 (NC- EST2019-ASR6H

**Fig. 3:**
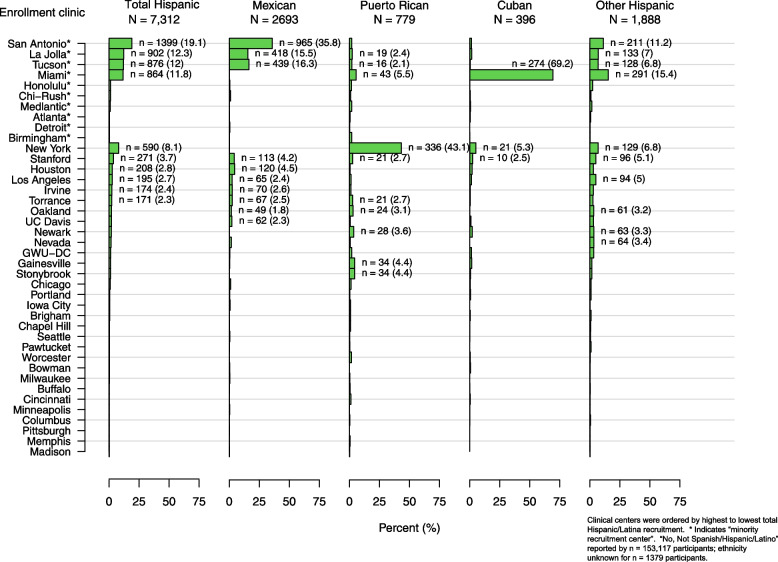
Distribution of WHI participants who identified as Hispanic/Latina by subgroups (*N* = 7312)

**Fig. 4:**
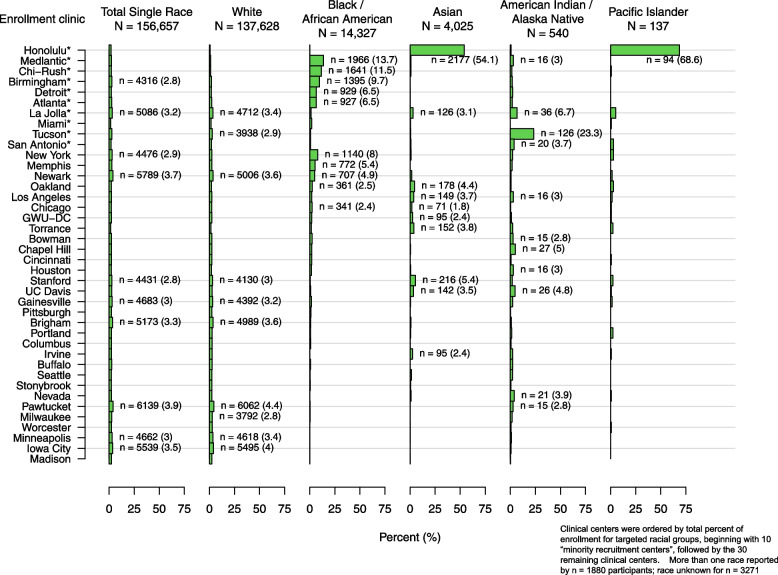
Distribution of WHI participants who identified as a single race by racial groups (*N* = 156,657)

One consequence of the strategy of designating “minority recruitment centers” to enhance racial and ethnic diversity was a potentially confounding influence of geographic and regional sociocultural factors on racial and ethnic comparisons. For example, among 7312 participants who identified as Hispanic/Latina (Table 2 and Fig. 3), the Miami site enrolled most of the Cuban WHI participants, the New York site enrolled the majority of the Puerto Rican participants, and the San Antonio (Texas), La Jolla (California) and Tucson (Arizona) sites enrolled most of the Mexican American participants, whereas other Texan and California sites enrolled fewer. Enrollment of women who identified as “other Spanish/Hispanic/Latina” was more variable. Similarly, any comparisons between “Asian” and “Pacific Islander” participants is confounded by the fact that 54% of the “Asian” and 69% of the “Pacific Islander” WHI participants were enrolled at the Honolulu (Hawaii) site and most resided on the island of Oahu, with most of the mainland Asians being enrolled by California WHI CCs (Fig. 4). On the other hand, the larger numbers and more even distribution of Black and White participants enrolled across the U.S. (Fig. 4 and Supplemental Figs. 1 and 2) offers an incredible opportunity to study the role of geographic region on health, including comparisons between Black and White WHI participants, taking into account differences by age and SDOH. Indeed, analyses are underway to explore differences and similarities across WHI race and ethnic groups and factors associated with structural racism and biases, taking into account differences associated with WHI geographic regions, e.g. Northeast, Southeast, Midwest and West. *(*See Supplemental Figs. 1, 2, 3, 4, 5, 6 for the distribution of each race (Figs. 1, 2, 3, 4, 5) and ethnic (Fig. 6) group by WHI Clinical Center).

## Specific considerations for including race and ethnicity in analyses

The Race and Ethnicity Task Force (R&E TF) created the WHI Race and Ethnicity Language and Data Interpretation Guide. The R&E TF was comprised of diverse members of the WHI community: the WHI Race, Ethnicity and Health Equity Special Interest Group, WHI investigators, analysts, and researchers whose research focused on race, ethnicity, health equity, social determinants of health, health disparities, and/or were themselves members and stakeholders of minoritized communities.

The WHI R&E TF recognizes that the concepts, terms and ideas in the WHI Race and Ethnicity Language and Data Interpretation Guide will continue to evolve and have recommended that it be reviewed and updated periodically, to reflect contemporary thinking.

The WHI Race and Ethnicity Language and Data Interpretation Guide (WHI website link) includes key points that would apply to most study cohorts:

- Develop Questions and Methodological Strategies Informed by Conceptual Frameworks [21]. In the study design and data interpretation stages of race- and ethnicity-focused research, identify conceptual models to target interpretation of the structural factors and racism underlying race and ethnic disparities. For example:
  - Public Health Critical Race Methodology (PHCR) [7, 8]offers conceptual guidance for distinguishing racism and health inequities from race as a risk factor.
  - “Scientists can consider using frameworks such as the National Institute of Minority Health and Health Disparities Research Framework [40] to develop study questions that consider domains of influence (e.g., behavioral, sociocultural/environmental) with levels of influence (e.g., individual, interpersonal, societal).” [21]
  - Those with limited expertise or experience with diverse populations should consider seeking this expertise and experience in the form of co-authors actively engaged in health disparities/ health equity research.
- Data Collection.
  - Characterization of racial and ethnic identity is not fixed and available options from national surveys (e.g., census) have changed over time and will continue to change. For example, WHI participants self-identified “race or ethnicity” at baseline as described above and self-identified ethnicity and race, per Census 2000 categories *(albeit with Asian and Pacific Islander subgroups presented as “race” categories)*, in 2003; however, as many participants were no longer active, WHI mapped baseline categories onto the 2003 categories (Fig. 2, Table 2). Terms which persons within each ethnic and race group identify with should be presented, with write-in options, and how these are combined for reporting or analyses should be carefully considered.
- Reporting of demographic data on race and ethnicity
  - Manuscripts should include an explanation of who identified participant race and ethnicity and the source of the classifications used (e.g. in WHI, this was by self-report).
  - Rationale for use of race as a key variable: For papers and ancillary studies where race is the primary exposure of interest or where analyses are stratified by race and/or ethnicity, authors should provide a clear, written definition and rationale for why race is being used (e.g., what it is serving as a proxy for).
- Data Analyses, Interpretation & Reporting
  - The term “other” has often been used as a “convenience” grouping or label for comparisons in data analysis when sample sizes for a given group are small compared to a dominant group, such as non-Hispanic Whites in most U.S. cohorts; however, combining “all other race and ethnic groups” has no valid scientific rationale and is clearly not informative across individual races or ethnicities and should, therefore, not be done. While there is considerable value in examining associations within select historically marginalized race and ethnic groups, the decision to make comparisons between race or ethnic groups should be informed by the research questions. Comparisons of race and ethnic groups to Non-Hispanic Whites by investigators should not be required. However, when highlighting the heterogeneity and resilience available within racial and ethnic groups, within heritage group analyses are recommended.
  - Authors are encouraged to address how representative of the reference population a given cohort is, in the context of interpreting the generalizability of the analytical results. For example, when evaluating the context of results from WHI that includes a range of race and ethnic, socioeconomic, and/or educational subgroups, it is important to consider generalizability along with relevant confounders and mediators for women aged 50–79 at baseline (1993–1998) and ages 70 and over now (2021) by race and ethnicity*,* i.e. *based on the proportion of older women within each race and ethnic group*. Discussion sections should address implications for analyses examining racial/ethnic inequities, which may be underestimated compared to those observed in the general U.S. older female population.
- Statistical power for race and ethnicity subgroup analyses:
  - We have an ethical responsibility to present data on all race and ethnic sub-groups, but appropriate interpretation is important. As is the case for all subgroup analyses, race and ethnicity subgroup analyses should be sufficiently powered to detect differences by that group. Results from analyses with insufficient power, based on smaller sample size, should be reported with caution. When describing results across race and ethnicity groups, it is essential that authors provide a clear context for interpretation and for applicability to any subgroup. The discussion should clearly acknowledge that sample selection limits interpretation of findings to the overall U.S. population or country of origin or heritage group identified in the manuscript.
- Retention by Race and Ethnicity:
  - Over time, sample composition of any given cohort will be influenced by selective drop-out that can be investigated through the use of inverse probability weighting and other methods. As noted above, WHI is currently analyzing known differences in retention by race and ethnic groups, recognizing inequality across all variables but also similarities compared to other women in their age range.

## Discussion

Race and ethnicity are clearly important variables that should be collected to describe the population, but as both serve as a proxy for both historical and ongoing disadvantage in social, economic, environmental and structural factors arising from racism, considerable caution should be applied when discussing their relationships to disease risk or to support recommendations regarding medical treatment [41]. Scientists should responsibly designate individuals as multidimensional beings exposed to differential life influencing factors that contribute to disease risk [42]. For example, underlying structural racism contributed to policies resulting in unequitable distribution of wealth, housing, health insurance and education, which has subsequently placed many racial and ethnic groups at higher risk for COVID-19 [41, 43].

Based on lessons learned and drawing on responsible research practices, current WHI Race and Ethnicity Language and Data Interpretation Guide recommend that:

- Studies be designed with inclusion of all populations in mind; assured access to research centers or removal of barriers to participation, as well as promotion of analytical methods, including mixed-methods, to better understand these factors as interventions are designed.
- Researchers actively, purposefully and with cultural-relevance, commit to recruiting a diverse sample for all research seeking to improve health.
- The scientific community should meaningfully commit to training the next generation of diverse scientists and research staff.
- Authors should develop clear direction and rationale for manuscripts that include scientific hypotheses with regards to race and ethnicity as proxies for social determinants of health and racism.
- Principal investigators should collect robust data on race and ethnicity, as well as intersections of religion, immigration status, country of origin, acculturation and the social determinants of health to inform research.
- Authors should clearly define the concepts and context of race and ethnicity as proxies for social determinants of health and racism in describing the purpose of the research and related manuscripts.
- Journal editors should require appropriate language and descriptors be included in manuscripts to robustly describe the population of interest.

Finally, the scientific and medical communities should define race within a robust historical, political, and contemporary cultural framework. This will advance scientific understanding of racism as it impacts health and wellness, and how it can be effectively dismantled. Race, when considered as a biological construct, perpetuates White supremacy in medicine and shifts focus from the fundamental causes of such differences, thereby impeding ability to effect meaningful change in understanding how systems and structures affect health [20, 44]. Greater detail, including country of origin, religion, immigration status and acculturation measures, combined with other social determinants of health, would be required to accurately enhance the rigor of research across every race and ethnic category in the WHI.

## Conclusion

There is a strong rationale for including race and ethnicity in health research such as longitudinal studies, like the Women’s Health Initiative. Race and ethnicity need to be clearly defined in testing health-related hypotheses as a social, not biological construct. Furthermore, National Institutes of Health (NIH)-funded cohorts such as the WHI should likewise extend to all participants across the nation the assurance of their commitment to report unbiased and rigorously quantified results intended to improve the health of all people groups.

## Supplementary Information

**Additional file 1:.**

**Additional file 2:.**

**Additional file 3:.**

**Additional file 4:.**

**Additional file 5:.**

**Additional file 6:.**

## Data Availability

The data that support the findings of this study are available from The Women’s Health Initiative but restrictions apply to the availability of these data, which were used under license for the current study, and so are not publicly available. Data are however available from the authors upon reasonable request and with permission of The Women’s Health Initiative.
