# Correction: Taking action to advance the study of race and ethnicity: the Women’s Health Initiative (WHI)

**DOI:** 10.1186/s40695-022-00083-w

**Published:** 2022-11-25

**Authors:** Lorena Garcia, Shawna Follis, Cynthia A. Thomson, Khadijah Breathett, Crystal Wiley Cené, Monik Jimenez, Charles Kooperberg, Kamal Masaki, Electra D. Paskett, Mary Pettinger, Aaron Aragaki, Peggye Dilworth-Anderson, Marcia L. Stefanick

**Affiliations:** 1 UC Davis School of Medicine, Department of Public Health Sciences, Davis, CA USA; 2 Stanford Prevention Research Center, Department of Medicine, Stanford University, Stanford, CA USA; 3 Department of Nutritional Sciences, University of Arizona, Tucson, AZ USA; 4 Division of Cardiology, College of Medicine, University of Arizona, Tucson, AZ USA; 5 UNC School of Medi- cine, Department of Medicine, Chapel Hill, NC USA; 6 Division of Women’s Health and Division of Preventive Medicine, Brigham and Women’s Hospital, Harvard Medical School, Boston, MA USA; 7 Public Health Sciences Division, Fred Hutchinson Cancer Research Center, Seattle, WA USA; 8 Department of Geriatric Medicine, John A. Burns School of Medicine, University of Hawaii, Honolulu, HI USA; 9 College of Public Health, The Ohio State University, Columbus, OH USA; 10 Department of Health Policy and Management, Gillings School of Global Public Health at UNC, Chapel Hill, NC USA; 11 Department of Obstetrics & Gynecology, Stanford University, Stanford, CA USA

**Correction: Women’s Midlife Health 8, 1 (2021)**

**https://doi.org/10.1186/s40695-021-00071-6**

Following publication of the original article [1], we have been notified that Fig. 1 is incorrect. It should be as per below:

**Fig. 1:**
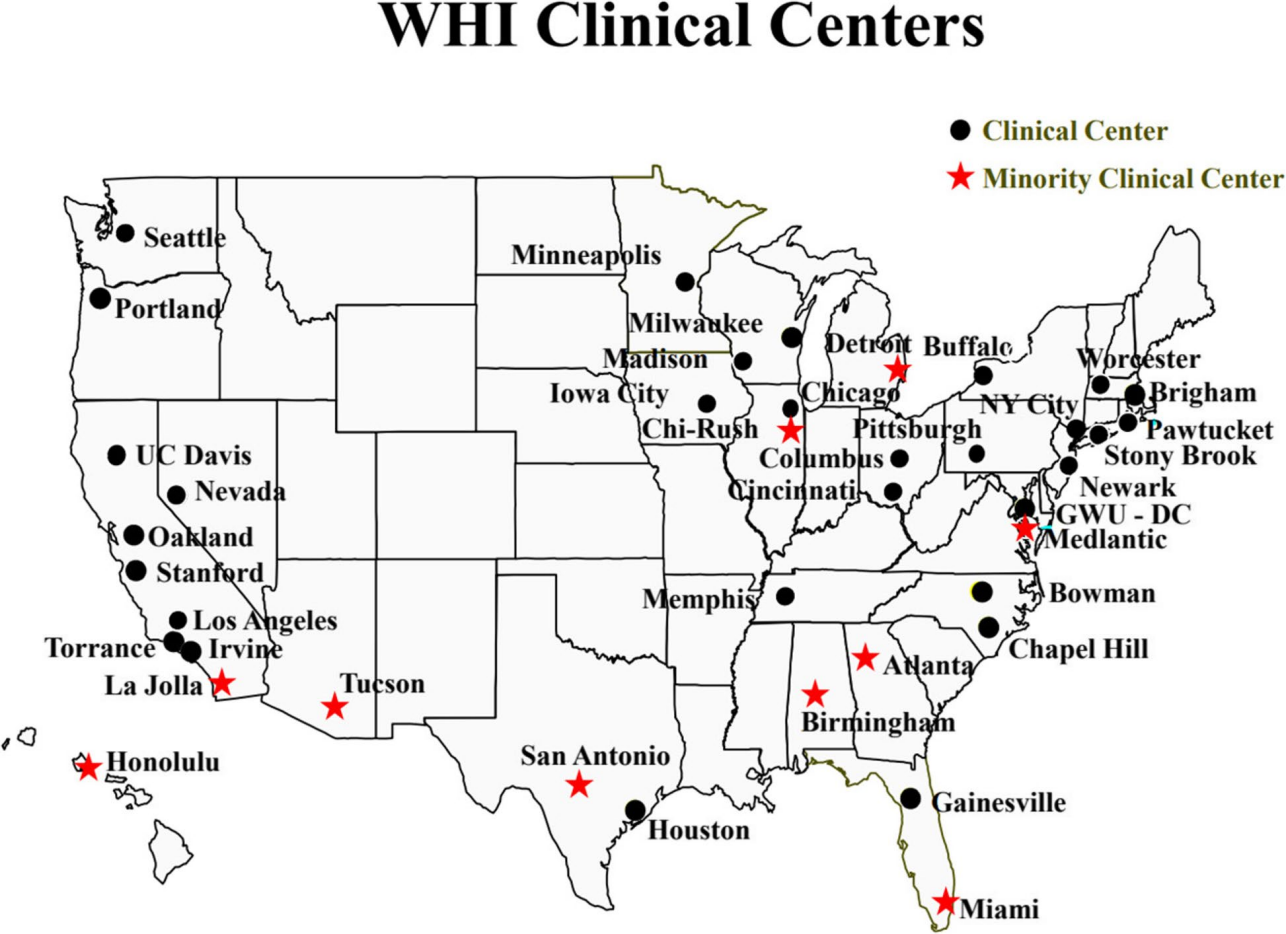
WHI Clinical Centers
