# Supplementary figures and images for: Taking action to advance the study of race and ethnicity: the Women’s Health Initiative (WHI)
Source: Womens Midlife Health. 2022 Jan 4;8:1. doi: 10.1186/s40695-021-00071-6 (PMC8724230; doi:10.1186/s40695-021-00071-6)

## Distribution of White participants (n = 137,628) by WHI clinic (%)

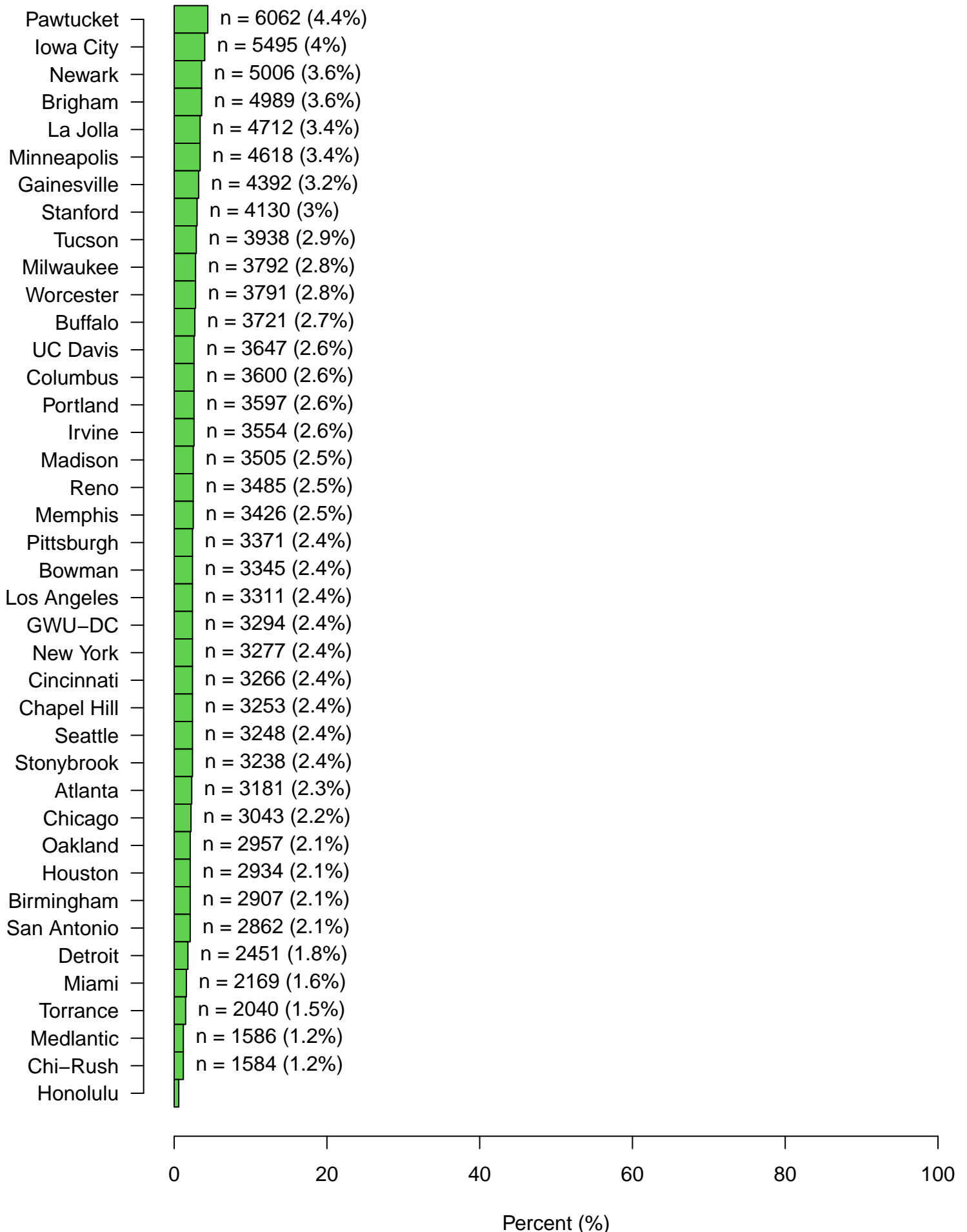

Supplement: Supplementary file 1 — Additional file 1:. [file 40695_2021_71_MOESM1_ESM.pdf]

## Distribution of Black participants (n = 14,327) by WHI clinic (%)

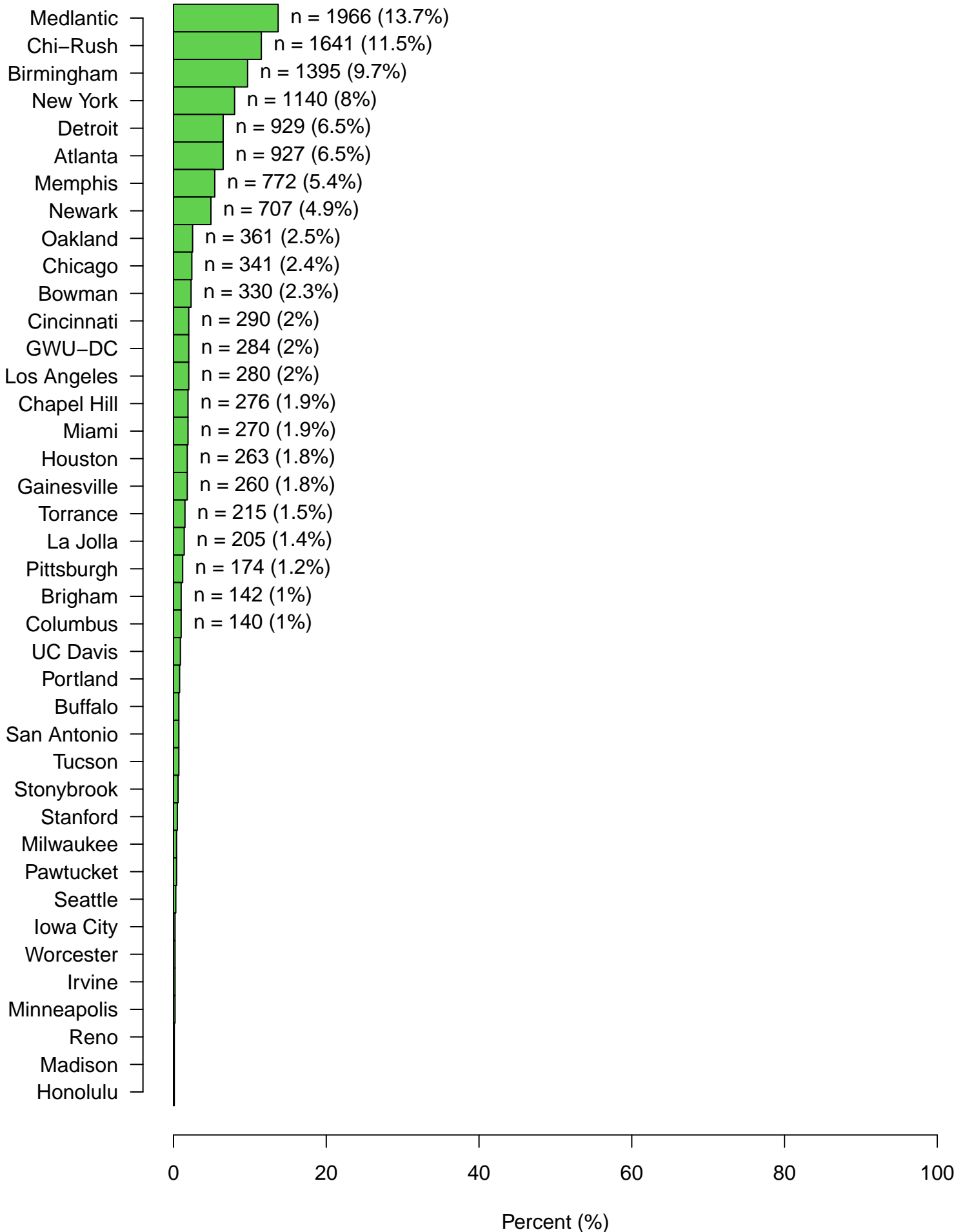

Supplement: Supplementary file 2 — Additional file 2:. [file 40695_2021_71_MOESM2_ESM.pdf]

## Distribution of Asian participants (n = 4025) by WHI clinic

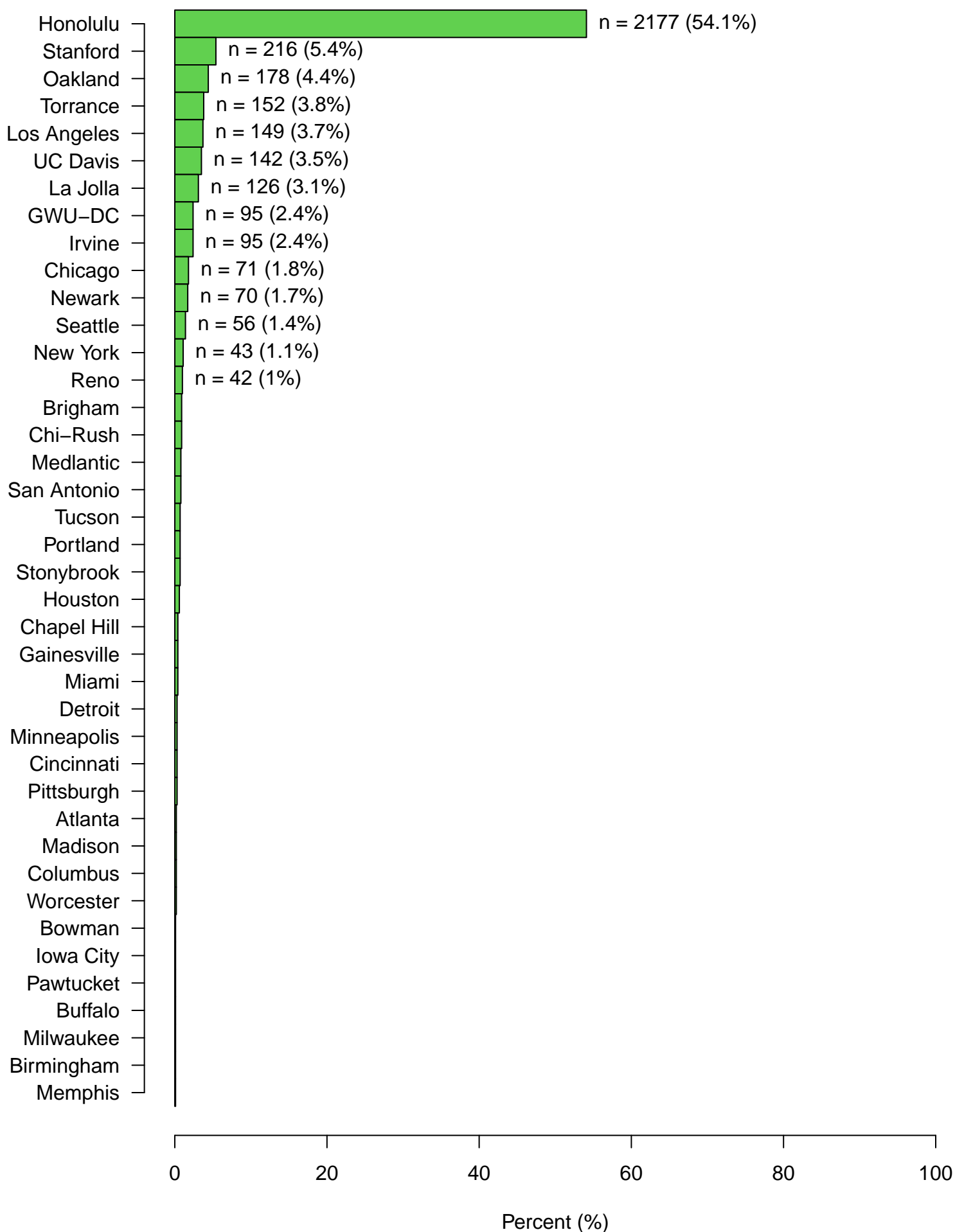

Supplement: Supplementary file 3 — Additional file 3:. [file 40695_2021_71_MOESM3_ESM.pdf]

## Distribution of American indian participants (n = 540) by WHI clinic (%)

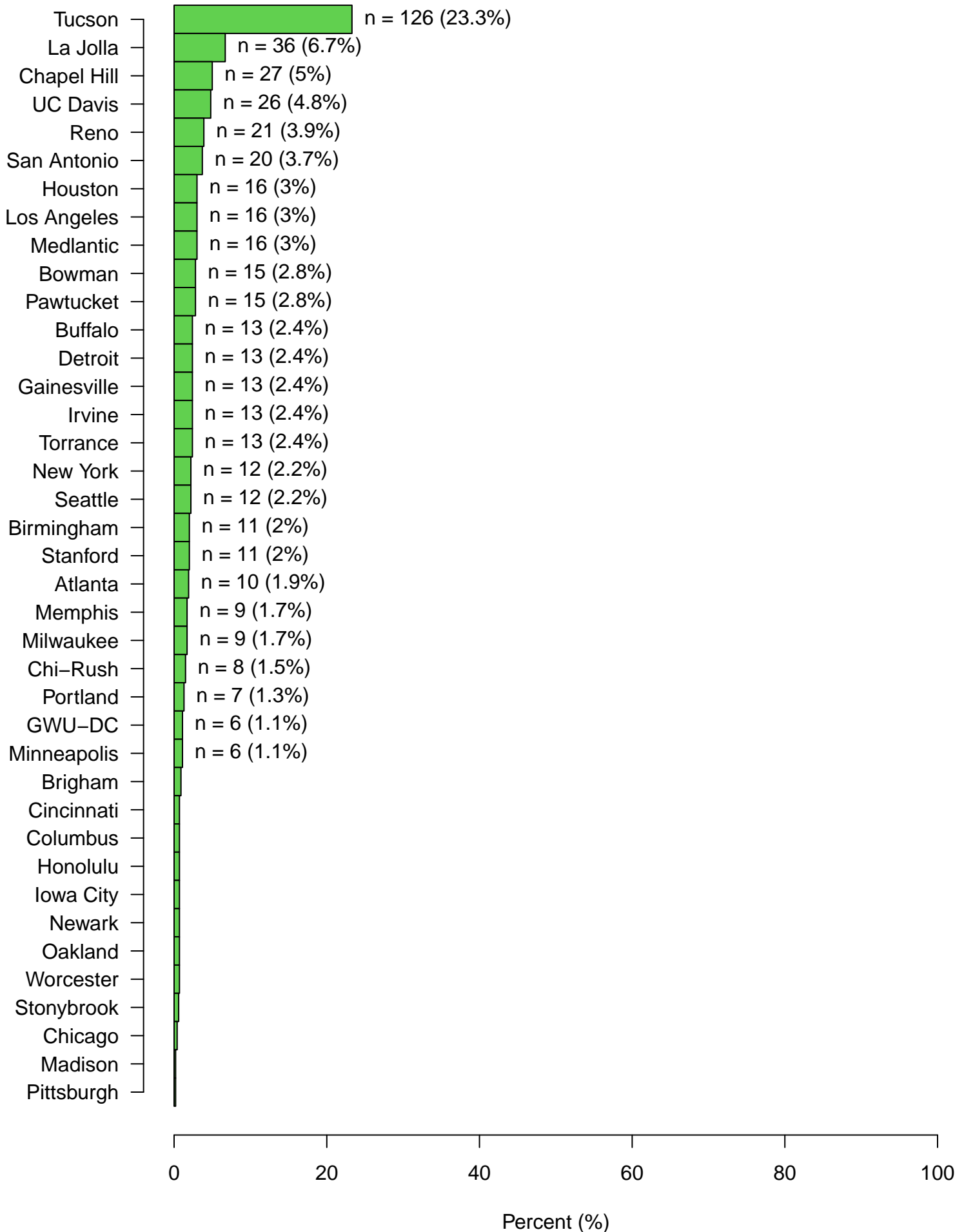

Supplement: Supplementary file 4 — Additional file 4:. [file 40695_2021_71_MOESM4_ESM.pdf]

**Distribution of Pacific Islander participants (n = 137) by WHI clinic (%)**

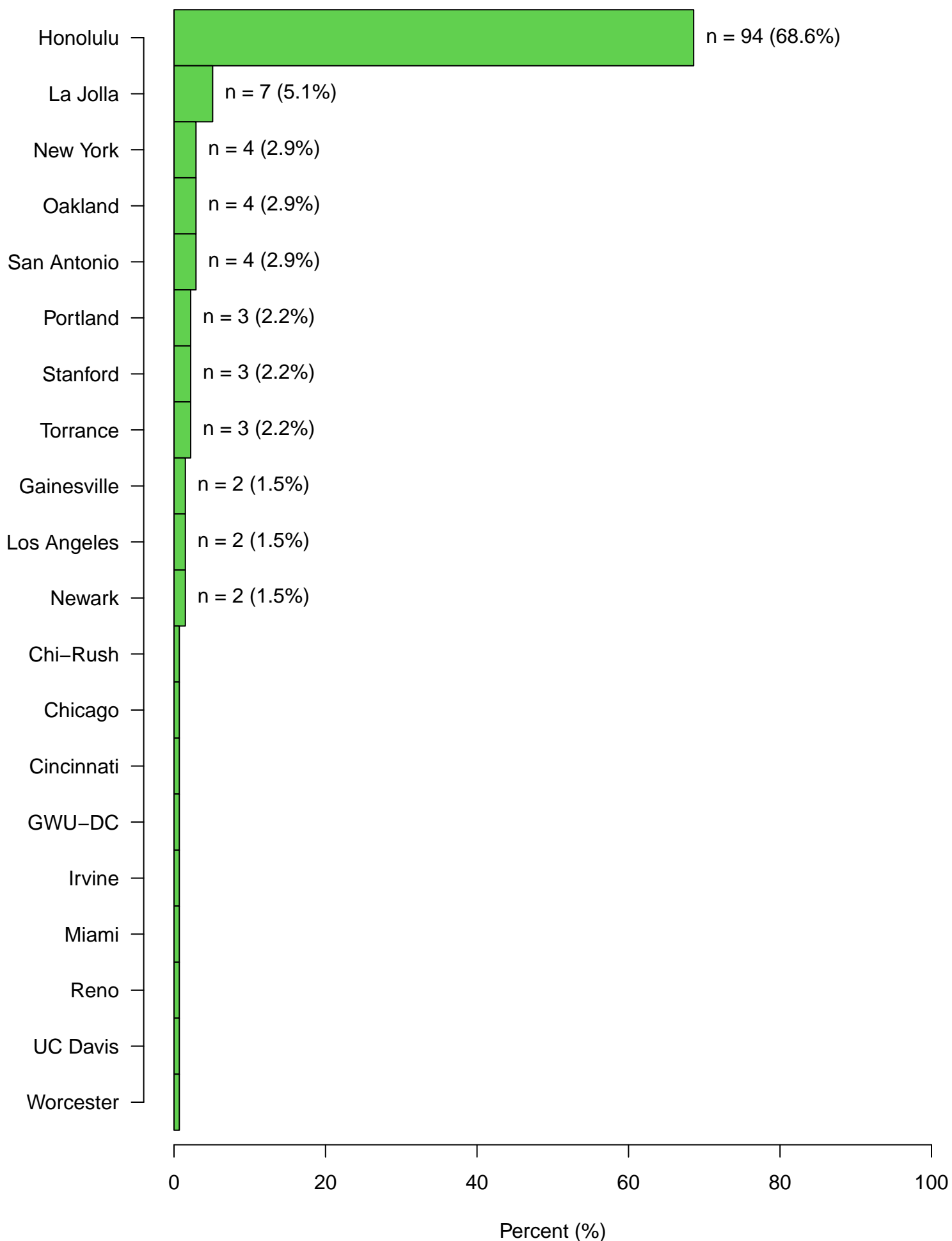

Supplement: Supplementary file 5 — Additional file 5:. [file 40695_2021_71_MOESM5_ESM.pdf]

## Distribution of Hispanic participants (n = 7312) by WHI clinic (%)

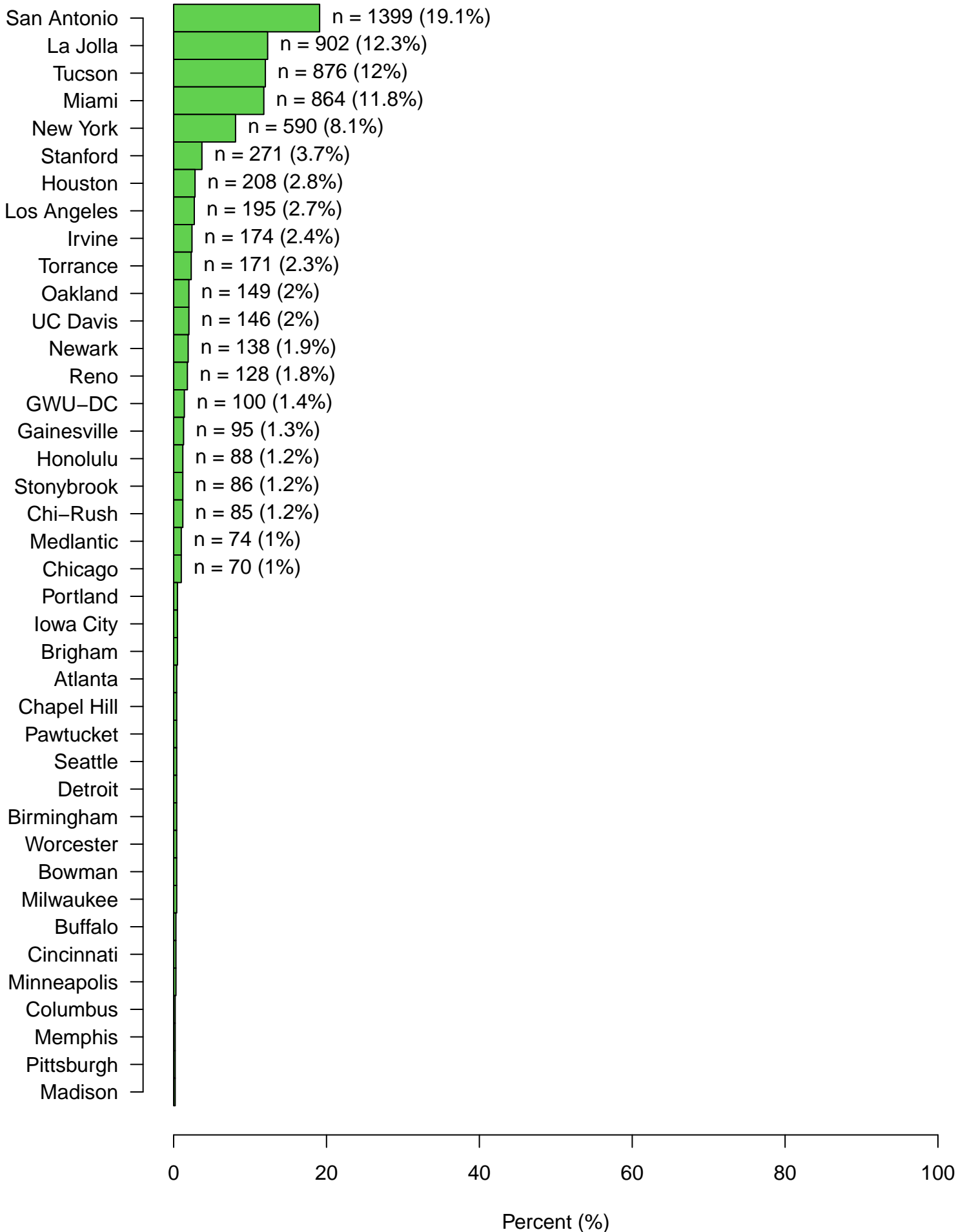

Supplement: Supplementary file 6 — Additional file 6:. [file 40695_2021_71_MOESM6_ESM.pdf]
